# Effects of an urban cable car intervention on quality of life: an observational, quasi-experimental study in Bogotá, Colombia (TrUST)

**DOI:** 10.1016/j.lana.2025.101126

**Published:** 2025-05-19

**Authors:** Laura Baldovino-Chiquillo, Olga L. Sarmiento, Donny S. Pasos, Leonardo Palencia-Pérez, Gary O'Donovan, Victor Cantillo-Garcia, Lina Martínez, Julian Arellana, Luis A. Guzman

**Affiliations:** a School of Medicine, Universidad de los Andes, Bogotá, Colombia; b Fundación Santa Fe de Bogotá, Bogotá, Colombia; c Department of Economics, Universidad de los Andes, Bogotá, Colombia; d School of Government, Universidad de los Andes, Bogotá, Colombia; e School of Engineering, Universidad de los Andes, Bogotá, Colombia; f Universidad ICESI, Cali, Colombia; g School of Engineering, Universidad del Norte, Barranquilla, Colombia

**Keywords:** Cable car, Transportation, Quality of life, Natural experiment, Latin America

## Abstract

**Background:**

There is little evidence about the relationships between transport interventions and quality of life in low-income settlements in Global South cities. The aim was to assess the effects of the TransMiCable cable car intervention on quality of life in males and females in a low-income settlement in Bogotá, Colombia.

**Methods:**

The Urban Transformations and Health (TrUST) natural experiment was conducted between 2018 and 2020 in intervention and control neighbourhoods. Overall quality of life, general health, and specific domains of quality of life were assessed in adults before and after the implementation of the TransMiCable using the World Health Organization's quality of life brief questionnaire. Adjusted multilevel linear regression models were used to estimate the effects on outcomes.

**Findings:**

Before the inauguration of TransMiCable, 2052 participants (1289 [62.8%] females and 763 [37.2%] males; mean age 43.5 years [SD 17.7]) completed the questionnaire. Analyses included 825 participants in the intervention group (80% of the baseline sample) and 854 participants in the control group (84% of the baseline sample) who completed the follow-up. Among females in the intervention area, there was an increase in quality of life (adjusted β for the time-by-group interaction, intervention *vs.* control: 5.81 points [95% CI: 2.47, 9.14]), and general health (adjusted β for the time-by-group interaction: 5.49 points [2.07, 8.92). Among males, quality of life and general health changes were not different in the intervention and control groups.

**Interpretation:**

Transport interventions, such as TransMiCable, could have meaningful impacts on the quality of life of women in low-income areas, promoting the achievement of sustainable development goals and improving well-being. A community-based, multisectoral approach is essential to designing integrated mobility policies that reflect the diverse needs of urban communities in the Global South.

**Funding:**

10.13039/100010269Wellcome Trust (as part of the Urban Health in Latin America project); Bogotá Urban Planning Department; Ministry of Science, Technology, and Innovation of Colombia; 10.13039/501100006070Universidad de Los Andes; Fundación Santa Fe de Bogotá; and 10.13039/501100004245Universidad del Norte.

**Research in context**

**Evidence before this study**

We searched PubMed and SciELO for articles published from inception up to December 2020, examining the association between the implementation of cable cars or new public transport systems and quality of life using the following search terms without language restrictions (“cable car” OR “ropeway” OR “transportation” OR “public transit”) AND (“quality of life”) AND (“impact” OR “effect”). We found evidence showing that sustainable urban planning, urban mobility, and urban environment can promote quality of life. In addition, previous qualitative studies in low-income settings in Latin America showed that cable cars can promote quality of life. However, there were no experimental or quasi-experimental studies evaluating the impacts of innovative urban transport interventions such as cable cars on health-related- quality of life and how these impacts differ among men and women, in low- and middle-income areas.

**Added value of this study**

This natural experiment provides novel evidence from one of the most populated cities in Latin America, showing innovative and integrated transport interventions, such as the TransMiCable cable car, can have meaningful impacts on the quality of life and general health perceptions of women in low-to-middle-income urban areas. These findings are encouraging in the context of achieving Sustainable Development Goals and promoting well-being.

**Implications of all the available evidence**

The results of this study may help guide the development of urban mobility policies that prioritize the holistic well-being, quality of life, and health of residents of low-to-middle-income areas while improving environmental sustainability. Regional and local policy makers should consider that women's life experiences and their access to the city public transport and public space are pivotal in their subjective well-being.

## Introduction

Quality of life, as defined by the World Health Organization, refers to how individuals perceive their position within their cultural context, including their goals, expectations, standards, and concerns.^1^ This concept encompasses aspects of an individuals’ physical, psychological, social, and environmental well-being.^1^^,^^2^ Therefore, it is crucial to understand how sustainable urban planning, mobility, and environment, can contribute to promoting quality of life and achieving the United Nations Sustainable Development Goals, particularly goals 3 “Ensure healthy lives and promote well-being” and 5 “Achieve gender equality and empower all women and girls”.^3^, ^4^, ^5^ Achieving these goals requires an integrated, multi-sectoral, and multi-stakeholder approach during the design and implementation of urban interventions.^5^^,^^6^

Cities in the Global South have made substantial investments in enhancing transportation systems, including innovative public transport services such as cable cars.^6^ TransMiCable in Bogotá, Colombia, is an aerial cable car with around 21,000 passengers daily that connects a once informal settlement on the city's outskirts with the Bus Rapid Transit system. Implementing this cable car was considered a more viable and sustainable alternative than tearing down homes and building roads.^7^ The TransMiCable intervention also includes broader urban improvements in the area such as the renovation of public parks and community centres. The design of this intervention was based on the principles of social urbanism, inspired by the progressive and innovative experience of urbanism in Medellín, Colombia,^8^ integrating the active participation of different urban sectors and communities. We have found that the TransMiCable intervention has helped people to maintain high levels of physical activity, especially walking for transport,^9^ while reducing exposure to air pollutants,^10^ and improving social capital,^11^ travel times, and transport satisfaction.^12^

There is limited research examining the relationship between transport interventions and quality of life definitions in socially disadvantaged communities with rapidly developing urban context.^13^^,^^14^ Previous studies of cable cars in low-income settings in Bogotá and Medellín in Colombia, and La Paz-El Alto in Bolivia, have shown that these transport systems can improve perceptions of quality of life.^12^^,^^13^^,^^15^, ^16^, ^17^ These cable cars have been associated with reduced travel times,^18^^,^^19^ improved access to social services,^19^ increased social cohesion,^20^^,^^21^ lower homicide rates,^21^^,^^22^ decreased unemployment,^19^ higher resident perceptions of inclusion and self-steem.^23^^,^^24^

However, there is limited experimental and quasi-experimental evidence about the effects of cable cars on health-related- quality of life, particularly in terms of how these impacts differ among demographic groups, such as men and women, in low- and middle-income areas. This understanding is crucial, especially in Latin America where about one-quarter of the urban population resides in informal settlements^25^ and where men and women often have different roles. Residents in these areas face multiple disadvantages and inequalities that affect their health and quality of life.^25^ Recent studies in Latin America have highlighted the role of gender differences in understanding these social and health inequalities.^26^^,^^27^ Women in these informal areas often fulfil responsibilities as single parents and hold informal jobs.^28^ In Bogotá, over 41% of the working population is engaged in informal work, with women comprising 41%.^29^ Around 10% of men and 35% of women in Bogotá dedicate at least 6 h daily to unpaid housework and care work.^30^ The percentage of carers who report poor or average health is twice as high as non-carers.^30^ Moreover, women's access to the city's resources and opportunities is often limited due to significant transport barriers, which can impact their travel options compared to men.^31^, ^32^, ^33^ Hence, it is reasonable to suggest that urban transport interventions could lead to greater increases in the quality of life of women in challenging socioeconomic contexts.

The aim of this study was to investigate the impacts of the TransMiCable cable car intervention on the quality of life of men and women in a low-income community on the outskirts of Bogotá. We hypothesised that the implementation of TransMiCable could improve the quality of life of residents around the area of influence of the cable car by improving transport conditions, the surrounding built environment, and access to services and facilities. This large, well-conducted study provides novel evidence from one of the most populated cities in Latin America, contributing to policy debates on the health and quality of life benefits of innovative and integrated transport interventions in low-income settlements in the Global South.

## Methods

### Study setting

The study Urban Transformations and Health (in Spanish: *Transformaciones Urbanas y Salud,* TrUST) of the Urban Health in Latin America (SALURBAL) project was conducted in two administrative areas of Bogotá: Ciudad Bolívar (intervention group) and San Cristóbal (control group). Both are characterized by rapid and unplanned urbanization, self-built settlements on steep hillsides, limited access to public services and transport, low socioeconomic levels, and significant challenges related to crime and violence.^34^

The TransMiCable cable car was inaugurated in December 2018 in Ciudad Bolívar. It has one line of 3.43 km in length, four stations, and 163 cabins, mobilizing more than 7.5 million passengers in the first year after its implementation. Moreover, the cable car was the main component of a broader urban development program that included renovated parks, a local history museum, a library, community centres, local market facilities, a tourism office, a citizen service office, a program to support physical improvements to homes, and a project to reduce landslides.^34^

In the TrUST study, baseline measurements in adults in the intervention and control areas were conducted from February 1, 2018, to December 18, 2018, before the TransMiCable implementation. Follow-up assessments in the intervention and control areas were collected after the opening of TransMiCable, from July 2, 2019, to March 15, 2020.

### Study design

**Fig. 1:**
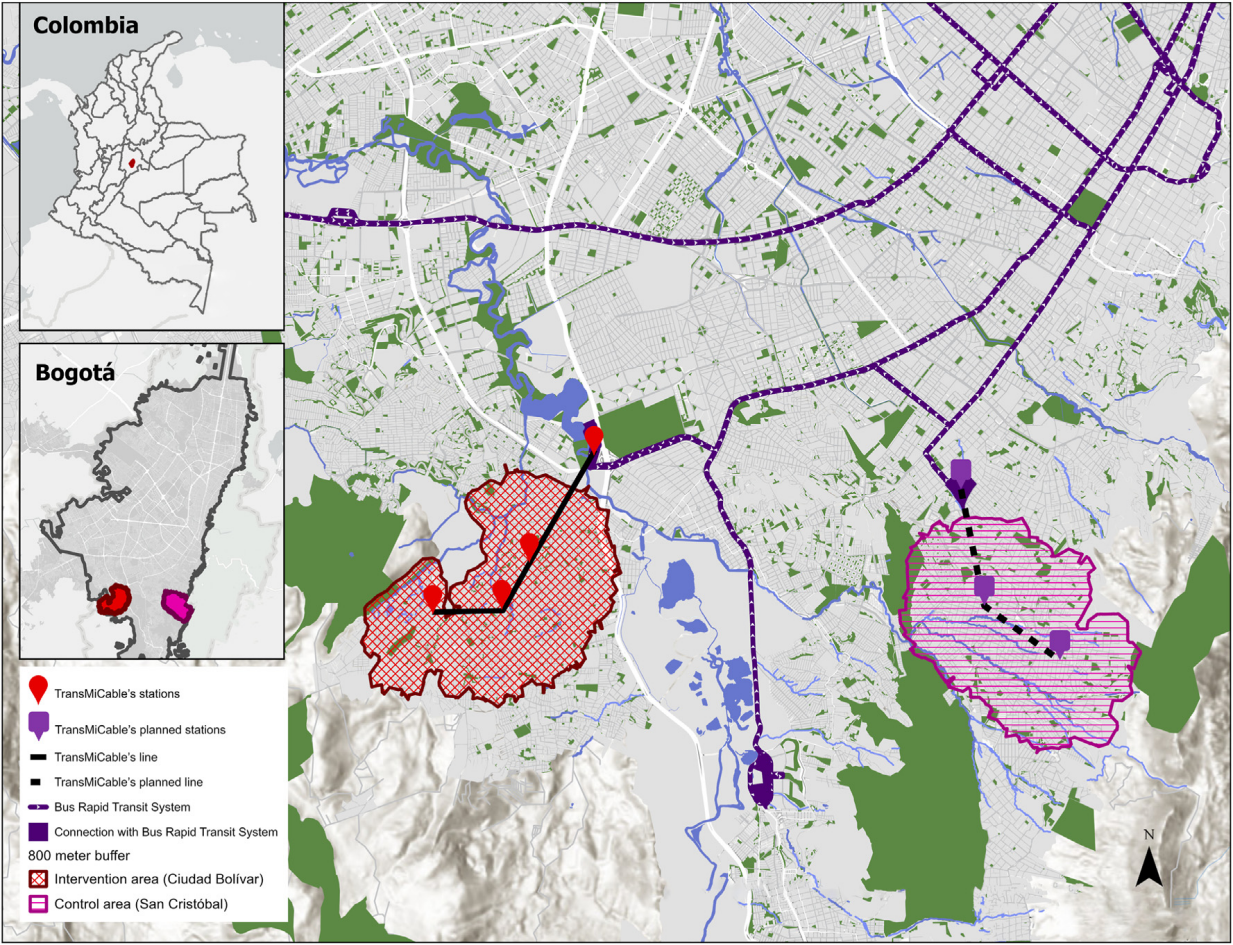
Map of the locations of intervention and control areas of the TrUST study in Bogotá, Colombia. The red-shaded area represents the influence area around the existing TransMiCable stations (Ciudad Bolívar Locality). The purple-shaded area corresponds to the control area around the projected stations of the new cable car system (San Cristóbal Locality). The map also shows the location of these two study areas within the city of Bogotá and the city's position within Colombia.

TrUST is an observational, quasi-experimental study with a mixed-methods approach described previously.^34^ Here, we focus on presenting the results of the quantitative analyses of the impacts of TransMiCable on quality of life. The intervention group comprised households within an 800-m buffer of each TransMiCable station in Ciudad Bolívar. The control group comprised households within an 800-m buffer of the potential locations for a new cable car in San Cristóbal projected for 2026. The intervention and control areas have similar topographical characteristics and are separated by around 4.8 km of mountainous land, with different transport routes and bus stations (Fig. 1).

### Participants

The study population comprised adults aged 18 years and older, who had lived in the intervention or control areas for at least 2 years, and who were not planning to move out of the study area within the next two years.^34^ We conducted a multi-stage sampling design. First, we selected 225 blocks from the intervention area, and 228 blocks from the control area with probability proportional to the density of the parcels in each block. Second, we systematically selected every third household within each block; third, we selected one adult using a random number table within each household. Everyone who participated in the study was given an incentive (a tote bag and a US$3 gift card) and was included in a draw for a US$150 gift card.

The study was approved by the ethics committee of the Universidad de Los Andes (record numbers 806–2017, 977–2019, 994–2019). All participants provided written informed consent.

### Measurements

Quality of life among participants was assessed using the World Health Organization's Quality of Life Brief Instrument (WHOQOL-BREF)^1^ in a household survey. Trained interviewers surveyed participants in their households. This instrument has been used in the Americas and Colombia, demonstrating that it provides a reliable and valid tool for assessing quality of life.^35^ The WHOQOL-BREF is a 26-item questionnaire consisting of one item for overall quality of life (*how would you rate your quality of life?),* one item for general health (*how satisfied are you with your health?),* and 24 items for four domains of quality of life: physical health (seven items on pain, dependence of medications, energy, mobility, sleep, daily living activities, and work capacity), psychological (six items on self-image, negative feelings, positive feelings, self-esteem, spirituality, and learning and concentration), social relationships (three items on personal relationships, social support, and sex life), and environment (eight items on safety, healthy environment, financial resources, opportunities to acquire new knowledge, leisure activities, physical environment, health services, and transportation). Each item of the WHOQOL-BREF was rated on a five-point Likert scale over the past two weeks. Domain scores were computed by averaging all items included in each domain and subsequently multiplying by a factor of four. The scores were then converted to a scale of 0–100.

Sociodemographic characteristics, transport mode, and travel time were assessed within the household survey.^34^ We collected data on the self-reported sex of the study participants through this questionnaire. All participants fell into the categories of 'females' or ‘males’. The road network distance between the participant's household and the nearest bus rapid transit station was estimated with a shortest path algorithm, according to the household and station geolocations, using the ArcGIS software (version 10.3).

### Statistical analysis

The sample size of individuals was powered to detect changes equivalent to standardized mean differences (d) in outcomes that range from 0.3 to 0.4. We, therefore, aimed to achieve a sample size of 800 adults in each group with a response rate at follow-up of 70%, which will provide a power of ≥80%.

First, descriptive statistics, including means, standard deviations, absolute and relative frequencies, were computed for sociodemographic, transport, and built environment variables, and each domain of quality of life within the intervention and control groups. Second, quality of life scores before and after the cable car's inauguration within the intervention and control groups were compared using paired T-tests. Third, effect sizes within each group were calculated using Cohen's d. Effect sizes of at least 0.20 to 0.50 were considered meaningful in the context of achieving sustainable development goals and delivering well-being.^36^ Finally, effects on quality of life were evaluated by comparing changes in the intervention group to those in the control group using multilevel linear regression models with random intercepts for individuals. The models featured the effects of time (before/after), group (intervention/control), and a time-by-group interaction to gauge the impact on quality of life. All models were adjusted for potential confounders, including age, sex, occupation, marital status, education, and distance to the Bus Rapid Transit station at baseline. All analyses included those participants with baseline and follow-up data and were stratified by sex. The model equation is described in the Appendix (p. 2). Additionally, the internal consistency of each domain of the WHOQOL-BREF questionnaire was estimated with Cronbach's alpha coefficient. Analyses were conducted using STATA version 17 and R Studio version 3.3.2.

### Role of the funding source

The funders of the study had no role in study design, data collection, data analysis, data interpretation, or writing of the report.

## Results

**Fig. 2:**
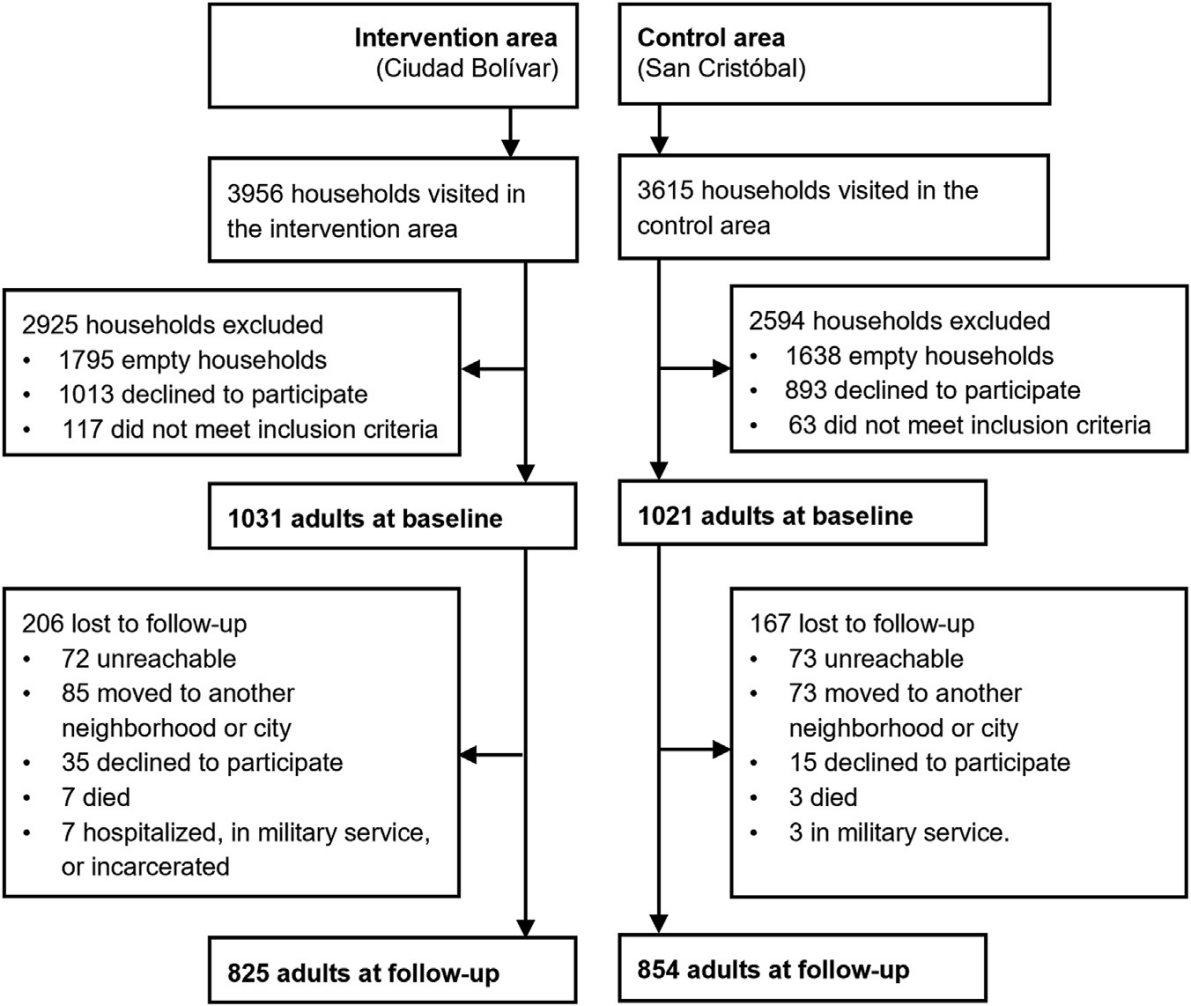
Sampling of participants. The TrUST study, 2018–2020. We invited one adult (18 years or older) per household who had lived in the intervention or control area for at least 2 years and were not planning to move within the next 2 years to participate in the study.

Household surveys were completed by 2052 adults (1031 in the intervention group and 1021 in the control group) before the inauguration of TransMiCable (Fig. 2). After the inauguration, 1679 adults completed the follow-up (825 [80.0%] adults in the intervention group and [83.6%] 854 in the control group). The individuals who completed baseline and follow-up WHOQOL-BREF questionnaire comprised the complete case sample for analyses.

The sociodemographic characteristics of participants at baseline were summarised previously (Table 1).^9^ The mean age of participants was 43.5 years (SD 17.7). Of the 2052 participants, 1289 (62.8%) were females and 763 (37.2%) were males. More than half of the individuals were married or partnered. Most participants attained elementary or high school education and reported a monthly household income of two or fewer minimum wage salaries. 1137 (55.4%) participants were working or studying. Public transport was the main transport mode for mandatory trips (travel for work or education). Before the inauguration of TransMiCable, the mean travel time (one way) was 110.0 min (SD 67.3) in the intervention group and 89.9 min (SD 54.1) in the control group. After the inauguration of TransMiCable, the mean travel time decreased to 90.2 min (SD 53.9) in the intervention area.

**Table 1:** Sociodemographic characteristics of participants and transport variables.

	Intervention (n = 1031)^a^	Control (n = 1021)^a^
**Sociodemographic characteristics**s		
Age, years	1031, 44.3 (18.1)	1021, 42.8 (17.3)
Sex		
Female	668 (64.8%)	621 (60.8%)
Male	363 (35.2%)	400 (39.2%)
Civil status		
Single	214 (20.8%)	331 (32.4%)
Married or with partner	551 (53.4%)	510 (50.0%)
Divorced, separated, or widowed	266 (25.8%)	180 (17.6%)
Education level		
Elementary or lower	399/1030 (38.7%)	256 (25.1%)
High school	466/1030 (45.2%)	514 (50.3%)
Technical, college, or graduate	165/1030 (16.0%)	251 (24.6%)
Monthly household income		
≤1 minimal wage (≤USD$264)	556/1023 (54.3%)	350/984 (35.6%)
>1 to ≤2 minimal wages (US$265–528)	386/1023 (37.7%)	485/984 (49.3%)
>2 minimal wages (≥US$529)	81/1023 (7.9%)	149/984 (15.1%)
Occupation		
Working	496/1029 (48.2%)	528/1019 (51.8%)
Studying	42/1029 (4.1%)	71/1019 (7.0%)
Household activities	322/1029 (31.3%)	294/1019 (28.8%)
Not working	169/1029 (16.4%)	126/1019 (12.4%)
**Transportation**		
Main transport mode for mandatory trips^a^		
Public	493/803 (61.4%)	554/823 (67.3%)
Public and active	174/803 (21.7%)	168/823 (20.4%)
Public and private	34/803 (4.2%)	21/823 (2.5%)
Public and informal	52/803 (6.5%)	12/823 (1.5%)
Private	50/803 (6.2%)	68/823 (8.3%)
Travel time for mandatory trips^b^, minutes	375, 110.0 (67.3)	396, 89.9 (54.1)

The TrUST study, Bogotá, 2018–2020.

Data are n, mean (standard deviation) or n (%).

a Participant numbers might not sum to the total group size due to missing data.

b Mandatory trips are defined as travel for work or education (one way). Public transport refers to state-owned modes of transport, such as local buses and the bus rapid transit system. Active transport refers to walking or cycling. Private transport refers to privately owned modes of transport, such as privately owned cars and motorbikes, or taxis. Informal transport refers to unofficial modes of transport that supplement public transport routes, such as unofficial local buses.

Quality of life scores at baseline were similar in the intervention and control areas (Table 2). However, females had lower quality of life scores than males in all the domains of quality of life in both areas, and there were different effects according to sex (Table 3).

**Table 2:** Changes in quality of life within the intervention and control groups before and after the inauguration of TransMiCable.

	Intervention				Control			
	Before [mean (SD)]	After [mean (SD)]	Change [mean (95% CI)]^a^	Effect size d^b^	Before [mean (SD)]	After [mean (SD)]	Change [mean (95% CI)]^a^	Effect size d^b^
Overall	n = 825				n = 854			
Overall quality of life	54.73 (24.17)	61.12 (25.00)	+6.39 (4.38, 8.40)	0.22	55.27 (21.11)	57.58 (23.85)	+2.31 (0.50, 4.12)	0.09
General health	55.82 (25.56)	59.64 (26.64)	+3.82 (1.88, 5.76)	0.13	56.88 (23.76)	57.29 (26.42)	+0.41 (−1.44, 2.26)	0.01
Domain 1. Physical health	66.65 (17.20)	65.82 (17.97)	−0.83 (−2.04, 0.38)	−0.05	65.07 (14.60)	67.48 (16.83)	+2.41 (1.27, 3.55)	0.14
Domain 2. Psychological	65.23 (16.73)	70.48 (16.85)	+5.25 (4.04, 6.47)	0.30	63.49 (13.72)	67.17 (14.99)	+3.69 (2.60, 4.77)	0.23
Domain 3. Social relationships	57.33 (20.35)	57.62 (20.12)	+0.28 (−1.32, 1.89)	0.01	54.28 (16.42)	57.54 (16.70)	+3.26 (1.91, 4.61)	0.16
Domain 4. Environment	51.81 (14.04)	56.10 (14.72)	+4.30 (3.19, 5.40)	0.27	51.80 (11.85)	55.57 (12.20)	+3.77 (2.87, 4.68)	0.28
Females	n = 540				n = 524			
Overall quality of life	53.06 (23.69)	60.28 (24.81)	+7.22 (4.75, 9.69)	0.25	54.15 (19.88)	55.49 (22.50)	+1.34 (−0.91, 3.58)	0.05
General health	51.53 (24.44)	56.20 (26.61)	+4.68 (2.20, 7.16)	0.16	53.44 (22.72)	52.67 (25.12)	−0.76 (−3.12, 1.59)	−0.03
Domain 1. Physical health	63.92 (16.83)	63.69 (17.71)	−0.22 (−1.76, 1.31)	−0.01	62.71 (14.23)	64.08 (16.15)	+1.37 (−0.11, 2.85)	0.08
Domain 2. Psychological	62.64 (16.60)	68.80 (16.90)	+6.17 (4.60, 7.73)	0.33	61.48 (13.29)	64.19 (14.27)	+2.70 (1.29, 4.12)	0.16
Domain 3. Social relationships	55.59 (20.46)	55.96 (20.59)	+0.37 (−1.67, 2.41)	0.02	52.78 (16.27)	54.91 (15.63)	+2.13 (0.38, 3.88)	0.10
Domain 4. Environment	50.89 (13.84)	54.97 (14.16)	+4.08 (2.74, 5.42)	0.26	50.44 (11.66)	53.72 (11.68)	+3.28 (2.12, 4.44)	0.24
Males	n = 285				n = 330			
Overall quality of life	57.89 (24.80)	62.72 (25.32)	+4.82 (1.36, 8.29)	0.16	57.05 (22.85)	60.91 (25.53)	+3.86 (0.82, 6.90)	0.14
General health	63.95 (25.71)	66.14 (25.49)	+2.19 (−0.89, 5.27)	0.08	62.35 (24.36)	64.62 (26.80)	+2.27 (−0.72, 5.27)	0.08
Domain 1. Physical health	71.84 (16.70)	69.86 (17.79)	−1.98 (−3.94, −0.02)	−0.12	68.81 (14.42)	72.88 (16.49)	+4.07 (2.29, 5.84)	0.25
Domain 2. Psychological	70.15 (15.88)	73.67 (16.31)	+3.52 (1.64, 5.40)	0.22	66.67 (13.81)	71.92 (14.90)	+5.25 (3.58, 6.92)	0.34
Domain 3. Social relationships	60.64 (19.75)	60.76 (18.84)	+0.12 (−2.48, 2.71)	0.01	56.67 (16.40)	61.72 (17.50)	+5.05 (2.93, 7.17)	0.26
Domain 4. Environment	53.55 (14.26)	58.26 (15.53)	+4.70 (2.74, 6.67)	0.28	53.96 (11.84)	58.51 (12.45)	+4.55 (3.09, 6.02)	0.34

The TrUST study, 2018–2020.

Complete cases (those with baseline and follow-up data) were included in the analyses.

a Data from paired T-test.

b Within group effect size from d Cohen coefficient.

**Table 3:** Effect of the TransMiCable intervention on quality of life.

	Unadjusted multilevel linear regression model (Time by group interaction)			Adjusted multilevel linear regression model (Time by group interaction)^a^		
	β	95% CI	p	β	95% CI	p
Overall						
Overall quality of life	+4.08	(1.38, 6.78)	<0.01	+4.03	(1.33, 6.73)	<0.01
General health	+3.41	(0.73, 6.09)	0.01	+3.44	(0.76, 6.13)	0.01
Domain 1. Physical health	−3.24	(−4.90, −1.59)	<0.01	−3.25	(−4.91, −1.58)	<0.01
Domain 2. Psychological	+1.56	(−0.06, 3.19)	0.06	+1.58	(−0.06, 3.21)	0.06
Domain 3. Social relationships	−2.98	(−5.07, −0.88)	0.01	−2.97	(−5.07, −0.87)	0.01
Domain 4. Environment	+0.52	(−0.90, 1.95)	0.47	+0.51	(−0.92, 1.94)	0.49
Females						
Overall quality of life	+5.89	(2.56, 9.22)	<0.01	+5.81	(2.47, 9.14)	<0.01
General health	+5.44	(2.02, 8.85)	<0.01	+5.49	(2.07, 8.92)	<0.01
Domain 1. Physical health	−1.59	(−3.72, 0.53)	0.14	−1.60	(−3.73, 0.54)	0.14
Domain 2. Psychological	+3.46	(1.36, 5.57)	<0.01	+3.48	(1.37, 5.59)	<0.01
Domain 3. Social relationships	−1.76	(−4.44, 0.92)	0.20	−1.74	(−4.43, 0.95)	0.20
Domain 4. Environment	+0.80	(−0.97, 2.57)	0.37	+0.78	(−0.99, 2.55)	0.39
Males						
Overall quality of life	+0.96	(−3.63, 5.55)	0.68	+0.96	(−3.65, 5.57)	0.68
General health	−0.08	(−4.36, 4.20)	0.97	−0.08	(−4.38, 4.22)	0.97
Domain 1. Physical health	−6.05	(−8.68, −3.41)	<0.01	−6.05	(−8.69, −3.41)	<0.01
Domain 2. Psychological	−1.73	(−4.24, 0.78)	0.18	−1.73	(−4.25, 0.79)	0.18
Domain 3. Social relationships	−4.93	(−8.28, −1.59)	<0.01	−4.93	(−8.29, −1.58)	<0.01
Domain 4. Environment	+0.15	(−2.29, 2.59)	0.91	+0.15	(−2.30, 2.60)	0.91

The TrUST study, 2018–2020.

Complete cases (those with baseline and follow-up data) were included in the analyses.

a Multilevel linear regression models with random intercepts for individuals, adjusted by age, sex, occupation, marital status, education, and distance to the bus-rapid-transit system.

Regarding females, the overall quality of life in the intervention area was rated at 53.06 points (SD 23.69) at baseline and increased to 60.28 (SD 24.81) after the inauguration of TransMiCable (change 7.22 points [95% CI 4.75–9.69], p < 0.001; d = 0.25). In the control area, the overall quality of life among females was rated at 54.15 points (SD 19.88) before the TransMiCable inauguration and did not change afterwards (change 1.34 points [95% CI −0.91 to 3.58], p = 0.242; d = 0.05). The overall quality of life increased among females in the intervention area with the TransMiCable intervention compared to females in the control area (adjusted β for the time-by-group interaction, intervention *vs*. control group: 5.81 points [2.47–9.14]). Among females in the intervention area compared to those in the control area, there was also an increase in the scores for general health (adjusted β for the time-by-group interaction: 5.49 points [2.07–8.92]), and the psychological domain of quality of life (adjusted β for the time-by-group interaction: 3.48 points [1.37–5.59]). After the TransMiCable implementation, no significant changes were found in the physical health, social relationships, and environmental domains of quality of life among females in the intervention group compared to those in the control group.

Regarding males, the overall quality of life was rated at 57.89 points (SD 24.80) in the intervention area and 57.05 points (SD 22.85) in the control area at baseline. After the implementation of TransMiCable, the increase in the overall quality of life score among males in the intervention group (change 4.82 [95% CI 1.36–8.29], p = 0.022; d = 0.16) was not different from the increase observed in the control group (change 3.86 [95% CI 0.82–6.90], p = 0.041, d = 0.14; adjusted β for the time-by-group interaction 0.96 points [−3.65 to 5.57]) (Table 3). After the TransMiCable implementation, no significant changes were found among males in the intervention group compared to those in the control group in the scores for overall health, and the psychological and environmental domains of quality of life.

Table S1 in the appendix shows the scores for each item of the four domains of quality of life. Within the psychological domain among females, all items showed an increase in the score after the opening of the TransMiCable in the intervention group.

The internal consistency was acceptable for the physical health (Cronbach's alpha coefficient; α = 0.76), psychological (α = 0.73), and environment domains (α = 0.71), and was regular for the domain of social relationships (α = 0.57).

## Discussion

In the TrUST natural experiment, the first of its kind in Latin America, we investigated the effect of the cable car TransMiCable and its wider urban interventions on the quality of life among women and men in low-income settlements. Our key finding was a meaningful and positive effect on quality of life among women. Specifically, women in the intervention area reported a 6-point increase in overall quality of life and a 5-point increase in general health after the TransMiCable implementation. The present study may help inform policy makers about the potential benefits to quality of life of urban cable cars and other of innovative and integrated systems, especially in women in disadvantaged areas.

Our evaluation of the TransMiCable cable car intervention suggests that the benefits to quality of life are greater in women than in men. Previous studies have shown that women are more likely to report a lower quality of life than men, mainly in Global South contexts.^37^ For example, in Colombia, women are generally less satisfied and happy than men.^38^ These differences could be explained by environmental and social stressors.^39^ Women in Latin America face a double burden of household work, different kinds of violence in their homes and public spaces, and lower-quality jobs than men.^40^ Within-group comparisons in women in the intervention group in the present study showed that changes in overall quality of life, general health, and the environmental health domain were not just statistically significant but meaningful in the context of achieving sustainable development goals and delivering well-being, having effect sizes of at least 0.20.^36^

The meaningful improvements in women's perceived quality of life after TransMiCable's inauguration could be explained in part by the multiple co-benefits of this intervention. Other findings of the TrUST study showed that TransMiCable users highly valued the reduced travel time and the improved comfort and security in the cabins.^41^ The intervention also improved several liveability domains, specifically, the cable car and the reconfiguration of the public space impacted on increasing access to public parks, reducing perceived social stigma in the community, and decreasing perceived insecurity in the neighborhood.^7^ Additionally, travel time reductions allowed women to have more leisure time with better life satisfaction and allocate more time to education and paid work.^12^^,^^42^

Improving safety perceptions in the transport is essential for promoting quality of life among women considering that, in Latin America, using public transport often leads to feelings of insecurity. In Bogotá, for example, it is reported that 84% of women have faced episodes of sexual harassment while using public transport.^43^ Therefore, improving safety perception is a direct mechanism to shift negative emotional experiences. The 2023 World Happiness Report also underscores the strong connection between life satisfaction and the freedom to make life choices.^44^ Increased ratings in the psychological domain of quality of life among women in our study showed that the TransMiCable intervention positively impacted women's overall well-being perception, positive feelings, and sense of freedom and safety. Although TransMiCable and the broader urban intervention have contributed to reducing perceived insecurity in public spaces, levels remain notably higher compared to other areas of the city. This suggests that, while TransMiCable has enhanced several aspects of daily life for residents, much work remains to promote a safely access to services and facilities.

We hypothesize that the benefits to quality of life of a large-scale transport intervention are increased with proper community engagement and participatory processes facilitating dialogue between the community and public sector, a model evident in TransMiCable's area of influence, where many community leaders were involved in the design and evaluation of the TransMiCable cable car intervention, including several women.^7^ This participatory approach applied for designing, implementing, and maintaining transport interventions with broader urban and social transformations also appears to be key to the documented success of other urban transport interventions in low-income settings in Latin America such as Medellín, Colombia, and La Paz-El Alto, Bolivia. The active participation of women in the process may increase agency and empowerment, which are related to higher well-being and health outcomes.^45^ Furthermore, integrated urban transport systems, coordinated with other city services and infrastructure efforts, offer the potential for sustained medium- and long-term enhancements in quality of life. This type of initiative has the potential to support ongoing improvements in the quality of life in the cable car's influence area, particularly for caregivers, who are predominantly women.^46^

The results of our study highlight the importance of considering the quality of life impacts of urban mobility policies in Bogotá and the Global South. Regional policy makers should consider that women's life experiences and access to the city and the public space are pivotal in women's subjective well-being and health. Additionally, the perspective to approach policymaking from a broader fashion underscores the importance of actively involving a diverse array of stakeholders, including communities, academics, and governmental bodies in the design and implementation of mobility policies to ensure they reflect the diverse needs, values, and aspirations of the community.

Our study has strengths and limitations. To the best of our knowledge, this large natural experiment is the first study of its kind in Latin America. Although our study was large enough to detect meaningful changes in perceptions of quality of life and general health by sex, larger studies may be needed to determine the impacts on other quality of life domains. We did not collect data on race or ethnicity. Future studies are needed to analyse how the impacts on quality of life might differ by race or ethnicity. We are not aware of co-interventions in the study areas that might have impacted participants’ health and quality of life, such as public space improvements, park constructions, and government subsidies. This study used a validated assessment tool for quality of life. However, data were self-reported, which may result in recall bias, social desirability bias, and other biases.

In conclusion, TrUST showed that transport interventions, such as TransMiCable, with integrated urban transformations and active community participation could increase quality of life and overall health in disadvantaged populations, especially among women who are systematically affected by violence and lack of leisure time. These findings may help guide the development of urban mobility policies that prioritize the holistic well-being of residents and environmental sustainability.

## Contributors

LBC: conceptualisation, data curation, formal analysis, investigation, methodology, project administration, software, supervision, visualisation, writing—original draft, and writing—review & editing. OLS: conceptualisation, data curation, formal analysis, funding acquisition, investigation, methodology, project administration, resources, software, supervision, validation, visualisation, writing—original draft, and writing—review & editing. DSP: data curation, investigation, software, and writing—review & editing. LPP: data curation, formal analysis, software, and writing—review & editing. GOD: methodology, supervision, writing—original draft, and writing—review & editing. VCC: data curation, formal analysis, software, and writing—review & editing. LM: writing—original draft, and writing—review & editing. JA: conceptualisation, funding acquisition, methodology, and writing—review & editing. LAG: conceptualisation, funding acquisition, methodology, visualisation, and writing—review & editing. All authors had full access to all the data in the study and had final responsibility for the decision to submit for publication.

## Data sharing statement

The Urban Health in Latin America (SALURBAL) project welcomes queries from anyone interested in learning more about its dataset and potential access to data. Further information about the SALURBAL dataset is available on the SALURBAL project website (www.lacurbanhealth.org) or via email (salurbal@drexel.edu). After publication of this study, the study protocols, data dictionaries, and requested study data may be made available to interested investigators after they have submitted a proposal by email and signed a data use agreement with SALURBAL, and if their study proposal, developed in collaboration with SALURBAL investigators, is approved by the SALURBAL proposal and publications committee. Some data might not be available to external investigators because of data confidentiality agreements.

## Editor note

The Lancet Group takes a neutral position with respect to territorial claims in published maps and institutional affiliations.

## Declaration of interests

We declare no competing interests.

## Appendix A Supplementary data

Abstract in Spanish
