# Supplementary material for: Effects of an urban cable car intervention on quality of life: an observational, quasi-experimental study in Bogotá, Colombia (TrUST)
Source: Lancet Reg Health Am. 2025 May 19;47:101126. doi: 10.1016/j.lana.2025.101126 (PMC12145807; doi:10.1016/j.lana.2025.101126)
Supplement: Abstract in Spanish [file mmc1.docx]

**Editor note:** *This translation in Spanish was submitted by the authors and we reproduce it as supplied. It has not been peer reviewed. Our editorial processes have only been applied to the original abstract in English, which should serve as reference for this manuscript.*

**Efectos de la implementación de un cable aéreo urbano en la calidad de vida: un estudio cuasiexperimental observacional en Bogotá, Colombia (TrUST)**

Laura Baldovino-Chiquillo MSc, Prof. Olga L Sarmiento PhD, Donny S Pasos BSc, Leonardo Palencia-Pérez MD, Prof. Gary O’Donovan PhD, Victor Cantillo-Garcia PhD, Prof. Lina Martínez PhD, Prof. Julian Arellana PhD, Prof. Luis A Guzman PhD.

**Resumen**

**Introducción:** Existe evidencia limitada sobre las relaciones entre las intervenciones de transporte y la calidad de vida en asentamientos de bajos ingresos en ciudades del Sur Global. El objetivo del estudio fue evaluar los efectos de la implementación del cable aéreo TransMiCable en la calidad de vida de hombres y mujeres en un asentamiento de bajos ingresos en Bogotá, Colombia.

**Métodos**: El experimento natural Transformaciones Urbanas y Salud (TrUST) se llevó a cabo entre 2018 y 2020 en áreas de intervención y control. La calidad de vida, la autopercepción de salud y dominios específicos de la calidad de vida de los adultos fueron evaluados antes y después de la implementación del cable aéreo utilizando el cuestionario breve de calidad de vida de la Organización Mundial de la Salud. Se utilizaron modelos de regresión lineal multinivel ajustados para estimar los efectos en los desenlaces.

**Hallazgos**: Antes de la inauguración de TransMiCable, 2052 participantes (1289 [62·8%] mujeres y 763 [37·2%] hombres; edad promedio 43·5 años [DE 17·7]) completaron el cuestionario. Los análisis incluyeron a 825 participantes en el grupo de intervención (80% de la muestra inicial) y a 854 participantes en el grupo de control (84% de la muestra inicial) que completaron el seguimiento. Entre las mujeres del área de intervención, hubo un aumento en la percepción de calidad de vida (β ajustado para la interacción tiempo-grupo, intervención vs. control: 5,81 puntos [IC 95%: 2,47, 9,14]) y la salud general (β ajustado para la interacción tiempo-grupo: 5,49 puntos [2,07, 8,92]). Entre los hombres, los cambios en la calidad de vida general y la salud general no fueron diferentes entre los grupos de intervención y control.

**Interpretación**: Las intervenciones de transporte, como TransMiCable, pueden tener impactos significativos en la calidad de vida de las mujeres en áreas de bajos ingresos, fomentando la consecución de los Objetivos de Desarrollo Sostenible y promoviendo el bienestar. Un enfoque comunitario y multisectorial es esencial para diseñar políticas de movilidad integradas que reflejen las diversas necesidades de las comunidades urbanas del Sur Global.
