# Supplementary material for: Effects of an urban cable car intervention on quality of life: an observational, quasi-experimental study in Bogotá, Colombia (TrUST)
Source: Lancet Reg Health Am. 2025 May 19;47:101126. doi: 10.1016/j.lana.2025.101126 (PMC12145807; doi:10.1016/j.lana.2025.101126)
Supplement: Supplementary Table [file mmc2.docx]

**SUPPLEMENTARY MATERIAL**

**Effects of an urban cable car intervention on quality of life: an observational, quasi-experimental study in Bogotá, Colombia (TrUST)**

Laura Baldovino-Chiquillo MSc^1^, Prof. Olga L Sarmiento PhD^2^, Donny S Pasos BSc^3^, Leonardo Palencia-Pérez MD^4^, Prof. Gary O’Donovan PhD^5^, Victor Cantillo-Garcia PhD^6^, Prof. Lina Martínez PhD^7^, Prof. Julian Arellana PhD^8^, Prof. Luis A Guzman PhD^9^.

**Affiliations:**

^1^ School of Medicine, Universidad de los Andes, Bogotá, Colombia; Fundación Santa Fe de Bogotá, Bogotá D.C., Colombia. Email: l.baldovino10@uniandes.edu.co

^2^ School of Medicine, Universidad de los Andes, Bogotá, Colombia. Email: osarmien@uniandes.edu.co

^3^ Department of Economics, Universidad de los Andes, Bogotá, Colombia; School of Government, Universidad de los Andes, Bogotá, Colombia. Email: d.pasos@uniandes.edu.co

^4^ School of Medicine, Universidad de los Andes, Bogotá, Colombia. Email: l.palencia@uniandes.edu.co

^5^ School of Medicine, Universidad de los Andes, Bogotá, Colombia; Latin American Brain Health Institute (BrainLat), Universidad Adolfo Ibáñez, Santiago, Chile. Email: drgaryodonovan@gmail.com

^6^ School of Engineering, Universidad de los Andes, Bogotá, Colombia. Email: va.cantillo@uniandes.edu.co

^7^ Universidad ICESI, Cali, Colombia. Email: lmmartinez@icesi.edu.co

^8^ School of Engineering, Universidad del Norte, Barranquilla, Colombia. Email: jarellana@uninorte.edu.co

^9^ School of Engineering, Universidad de los Andes, Bogotá, Colombia. Email: la.guzman@uniandes.edu.co

**Corresponding author:** Olga L. Sarmiento, osarmien@uniandes.edu.co, Universidad de Los Andes, Carrera 1 N° 18A-12, Bogotá – Colombia, Phone: (57-1) 3394949 ext 3785

**Equation form for the multilevel linear regression models**

The form of the multilevel linear regression models is shown in the following equation:

$$Y_{it}= \beta_{0}+{\beta_{1}Time}_{it}+{\beta_{2}Treatment}_{i}+\beta_{3}\left( {Time}_{it}*{Treatment}_{i} \right)+ {\theta X}_{i}+ u_{i}+ e_{it}$$

Where:

$Y_{it}$ are the outcomes of quality of life for an individual $i$ that was observed during two periods $t$ *(*$t$ *=* T_0_, T_1_).

$\beta_{0}$ is the overall intercept (average across individuals).

${Time}_{it}$ is the period at which measurement $t$was taken on individual $i$. T_0_ denotes the pre-intervention period and T_1_ is the post-intervention period.

${Treatment}_{i}$ is the treatment condition dummy variable, equal to 1 for individuals living in Ciudad Bolivar (intervention group) and 0 for individuals living in San Cristóbal (control group).

${Time}_{it}*{Treatment}_{i}$ is the interaction term used to assess the effect of TransMiCable by comparing the changes in outcomes over time between the intervention and control groups.

$X_{i}$ are individual characteristics at baseline measurement (age, sex, occupation, education, marital status, and distance to the Bus Rapid Transit station).

$u_{i} \sim N\left( 0, \sigma_{u}^{2} \right)$ is an individual-specific random effect (between-individual variance in $y$).

$e_{it} \sim N\left( 0, \sigma_{e}^{2} \right)$ is a time-varying residual (within-individual variance $y$).

**Table S1.** Items of the World Health Organization’s quality of life brief questionnaire for each domain of quality of life before and after the inauguration of TransMiCable. The TrUST study, 2018-2020.

| **Domains and items** | **Question** | **Scale** | **Total** | | | | **Women** | | | | **Men** | | | |
| --- | --- | --- | --- | --- | --- | --- | --- | --- | --- | --- | --- | --- | --- | --- |
|  |  |  | **Intervention (N=825)** | | **Control (N=854)** | | **Intervention (N=540)** | | **Control  (N=524)** | | **Intervention (N=285)** | | **Control  (N=330)** | |
|  |  |  | **Before** | **After** | **Before** | **After** | **Before** | **After** | **Before** | **After** | **Before** | **After** | **Before** | **After** |
| **Domain 1. Physical health** | | |  |  |  |  |  |  |  |  |  |  |  |  |
| 1.1. Pain and discomfort | To what extent do you feel that (physical) pain prevents you from doing what you need to do? | 1:5 (an extreme amount - not at all) | 3.96 (1.19) | 3.80 (1.27) | 4.03 (1.15) | 3.97 (1.26) | 3.81 (1.20) | 3.66 (1.30) | 3.91 (1.20) | 3.80 (1.28) | 4.25 (1.12) | 4.06 (1.17) | 4.21 (1.06) | 4.24 (1.17) |
| 1.2. Dependence on medicinal substances and medical aids | How much do you need any medical treatment to function in your daily life? | 1:5 (an extreme amount - not at all) | 4.10 (1.19) | 4.02 (1.17) | 4.20 (1.16) | 4.11 (1.23) | 3.98 (1.21) | 3.90 (1.18) | 4.06 (1.22) | 3.96 (1.25) | 4.34 (1.10) | 4.25 (1.14) | 4.42 (1.03) | 4.35 (1.15) |
| 1.3. Energy and fatigue | Do you have enough energy for everyday life? | 1:5 (not at all - completely) | 3.50 (0.96) | 3.57 (0.98) | 3.41 (0.79) | 3.62 (0.94) | 3.39 (0.94) | 3.46 (0.97) | 3.32 (0.78) | 3.49 (0.90) | 3.70 (0.96) | 3.79 (0.98) | 3.55 (0.81) | 3.84 (0.95) |
| 1.4. Mobility | How well are you able to get around? | 1:5 (very poor - very good) | 3.85 (1.07) | 3.73 (0.96) | 3.58 (0.87) | 3.67 (0.89) | 3.79 (1.06) | 3.68 (0.97) | 3.52 (0.86) | 3.54 (0.84) | 3.96 (1.06) | 3.82 (0.93) | 3.67 (0.89) | 3.88 (0.91) |
| 1.5. Sleep and rest | How satisfied are you with your sleep? | 1:5 (very dissatisfied - very satisfied) | 3.31 (1.11) | 3.35 (1.05) | 3.24 (1.00) | 3.31 (1.10) | 3.16 (1.14) | 3.28 (1.07) | 3.13 (1.02) | 3.19 (1.09) | 3.59 (1.02) | 3.47 (1.01) | 3.40 (0.93) | 3.49 (1.09) |
| 1.6. Activities for daily living | How satisfied are you with your ability to perform your daily living activities? | 1:5 (very dissatisfied - very satisfied) | 3.50 (0.91) | 3.56 (0.92) | 3.44 (0.74) | 3.62 (0.88) | 3.39 (0.90) | 3.51 (0.91) | 3.37 (0.72) | 3.52 (0.86) | 3.71 (0.89) | 3.65 (0.93) | 3.55 (0.76) | 3.78 (0.89) |
| 1.7. Work capacity | How satisfied are you with your capacity for work? | 1:5 (very dissatisfied - very satisfied) | 3.45 (1.03) | 3.40 (1.08) | 3.33 (0.83) | 3.59 (1.07) | 3.38 (1.00) | 3.34 (1.04) | 3.24 (0.79) | 3.44 (1.08) | 3.57 (1.08) | 3.52 (1.16) | 3.47 (0.87) | 3.83 (1.02) |
| **Domain 2. Psychological** | | |  |  |  |  |  |  |  |  |  |  |  |  |
| 2.1. Bodily image and appearance | Are you able to accept your bodily appearance? | 1:5 (not at all - completely) | 3.90 (1.00) | 4.10 (0.96) | 3.67 (0.80) | 3.85 (0.86) | 3.82 (1.04) | 4.07 (0.98) | 3.62 (0.79) | 3.76 (0.85) | 4.05 (0.89) | 4.16 (0.93) | 3.77 (0.80) | 4.00 (0.85) |
| 2.2. Negative feelings | How often do you have negative feelings such as blue mood, despair, anxiety, depression? | 1:5 (always - never) | 3.46 (1.19) | 3.58 (1.23) | 3.74 (1.08) | 3.74 (1.15) | 3.29 (1.20) | 3.39 (1.25) | 3.60 (1.07) | 3.56 (1.17) | 3.78 (1.10) | 3.94 (1.11) | 3.95 (1.05) | 4.02 (1.07) |
| 2.3. Positive feelings | How much do you enjoy life? | 1:5 (not at all - an extreme amount) | 3.25 (1.08) | 3.53 (1.10) | 3.37 (0.83) | 3.48 (0.93) | 3.14 (1.08) | 3.47 (1.12) | 3.28 (0.82) | 3.36 (0.93) | 3.45 (1.05) | 3.64 (1.08) | 3.50 (0.83) | 3.65 (0.92) |
| 2.4. Self-esteem | How satisfied are you with yourself? | 1:5 (very dissatisfied - very satisfied) | 3.80 (0.94) | 3.94 (0.93) | 3.53 (0.78) | 3.84 (0.91) | 3.72 (0.95) | 3.90 (0.93) | 3.44 (0.79) | 3.73 (0.91) | 3.94 (0.91) | 4.02 (0.91) | 3.66 (0.76) | 4.02 (0.88) |
| 2.5. Spirituality/ religion/ personal beliefs | To what extent do you feel your life to be meaningful? | 1:5 (not at all - an extreme amount) | 3.84 (0.99) | 4.14 (0.94) | 3.59 (0.84) | 3.70 (0.90) | 3.77 (1.02) | 4.14 (0.97) | 3.54 (0.81) | 3.61 (0.88) | 3.97 (0.92) | 4.13 (0.90) | 3.68 (0.87) | 3.85 (0.91) |
| 2.6. Thinking, learning, memory, and concentration | How well are you able to concentrate? | 1:5 (not at all - extreme) | 3.41 (0.93) | 3.63 (0.90) | 3.34 (0.76) | 3.51 (0.80) | 3.29 (0.94) | 3.54 (0.90) | 3.27 (0.74) | 3.38 (0.75) | 3.64 (0.88) | 3.79 (0.89) | 3.44 (0.77) | 3.72 (0.82) |
| **Domain 3. Social relationships** | | |  |  |  |  |  |  |  |  |  |  |  |  |
| 3.1. Personal relationships | How satisfied are you with your personal relationships? | 1:5 (very dissatisfied - very satisfied) | 3.60 (0.97) | 3.68 (0.92) | 3.43 (0.78) | 3.67 (0.88) | 3.56 (0.99) | 3.62 (0.93) | 3.40 (0.76) | 3.61 (0.86) | 3.68 (0.94) | 3.79 (0.87) | 3.49 (0.81) | 3.78 (0.90) |
| 3.2. Sexual activity | How satisfied are you with your sex life? | 1:5 (very dissatisfied - very satisfied) | 3.20 (1.19) | 3.19 (1.20) | 3.05 (0.99) | 3.08 (1.07) | 3.09 (1.22) | 3.03 (1.25) | 2.92 (1.03) | 2.88 (1.03) | 3.40 (1.12) | 3.51 (1.03) | 3.24 (0.91) | 3.40 (1.07) |
| 3.3. Social support | How satisfied are you with the support you get from your friends? | 1:5 (very dissatisfied - very satisfied) | 3.08 (1.13) | 3.04 (1.12) | 3.04 (0.95) | 3.15 (0.91) | 3.01 (1.17) | 3.07 (1.15) | 3.02 (0.97) | 3.10 (0.94) | 3.19 (1.05) | 2.99 (1.06) | 3.07 (0.93) | 3.22 (0.86) |
| **Domain 4. Environment** | | |  |  |  |  |  |  |  |  |  |  |  |  |
| 4.1. Freedom, physical safety, and security | How safe do you feel in your daily life? | 1:5 (not at all - extreme) | 3.19 (0.95) | 3.45 (0.90) | 3.18 (0.73) | 3.23 (0.84) | 3.12 (0.91) | 3.42 (0.91) | 3.15 (0.70) | 3.11 (0.83) | 3.32 (1.00) | 3.50 (0.87) | 3.22 (0.78) | 3.42 (0.83) |
| 4.2. Home environment | How healthy is your physical environment? | 1:5 (not at all - extreme) | 2.99 (0.94) | 3.27 (0.92) | 3.17 (0.73) | 3.36 (0.77) | 2.94 (0.94) | 3.23 (0.93) | 3.14 (0.74) | 3.31 (0.76) | 3.08 (0.93) | 3.35 (0.90) | 3.23 (0.71) | 3.44 (0.78) |
| 4.3. Financial resources | Have you enough money to meet your needs? | 1:5 (not at all - completely) | 2.56 (0.94) | 2.74 (0.96) | 2.62 (0.82) | 2.83 (0.82) | 2.51 (0.94) | 2.69 (0.95) | 2.56 (0.80) | 2.78 (0.78) | 2.66 (0.92) | 2.84 (0.98) | 2.72 (0.83) | 2.92 (0.88) |
| 4.4. Opportunities for acquiring new information and skills | How available to you is the information that you need in your day-to-day life? | 1:5 (not at all - completely) | 3.13 (0.93) | 3.34 (0.85) | 3.27 (0.75) | 3.54 (0.85) | 3.10 (0.92) | 3.34 (0.82) | 3.23 (0.73) | 3.45 (0.82) | 3.18 (0.95) | 3.34 (0.91) | 3.33 (0.78) | 3.68 (0.89) |
| 4.5. Participation in and opportunities for recreation /leisure activities | To what extent do you have the opportunity for leisure activities? | 1:5 (not at all - completely) | 2.99 (1.10) | 3.08 (1.06) | 2.90 (1.05) | 3.16 (0.93) | 2.92 (1.10) | 3.00 (1.06) | 2.81 (1.07) | 3.08 (0.93) | 3.11 (1.08) | 3.24 (1.04) | 3.03 (1.00) | 3.28 (0.92) |
| 4.6. Physical environment | How satisfied are you with the conditions of your living place? | 1:5 (very dissatisfied - very satisfied) | 3.51 (0.98) | 3.55 (0.98) | 3.42 (0.80) | 3.61 (0.85) | 3.48 (0.98) | 3.48 (1.00) | 3.35 (0.80) | 3.55 (0.86) | 3.55 (0.96) | 3.69 (0.93) | 3.52 (0.79) | 3.71 (0.82) |
| 4.7. Health and social care | How satisfied are you with your access to health services? | 1:5 (very dissatisfied - very satisfied) | 3.81 (0.91) | 3.59 (0.95) | 3.52 (0.74) | 3.72 (0.79) | 3.83 (0.90) | 3.56 (0.93) | 3.47 (0.74) | 3.64 (0.79) | 3.78 (0.91) | 3.63 (0.97) | 3.61 (0.74) | 3.85 (0.78) |
| 4.8. Transport | How satisfied are you with your transport? | 1:5 (very dissatisfied - very satisfied) | 2.41 (0.99) | 2.93 (1.02) | 2.50 (0.98) | 2.33 (0.91) | 2.38 (0.94) | 2.86 (1.00) | 2.43 (0.95) | 2.28 (0.85) | 2.46 (1.09) | 3.05 (1.07) | 2.61 (1.02) | 2.42 (0.99) |

Our results do not show significant changes in the scores for the environmental and social relationships domains of quality of life. However, there are notable improvements in some of the indicators for each domain, especially in the satisfaction with transport, the built environment, and safety, which are components that affect the everyday life of women. Additionally, there was no apparent effect on some indicators related to access to health and education services, which might suggest that even though TransMiCable improves some aspects, there is still a lot to do to solve the community needs in these areas.
